# The Control of Novel and Traditional Elemental Impurities: Ag, Au, Co, Cs, Li, Mo, Se, Sr, and V in Mint Tea Infusions (Peppermint, *Mentha piperita* L.) Available in Poland: A Health Risk Assessment

**DOI:** 10.3390/ijerph192416564

**Published:** 2022-12-09

**Authors:** Justyna Milan, Adrian Frydrych, Maciej Noga, Elżbieta Kondratowicz-Pietruszka, Mirosław Krośniak, Kamil Jurowski

**Affiliations:** 1 Laboratory of Innovative Toxicological Research and Analyses, Institute of Medical Studies, Medical College, Rzeszów University, Aleja Majora W. Kopisto 2a, 35-959 Rzeszow, Poland; 2 Department of Regulatory and Forensic Toxicology, Institute of Medical Expertises, Aleksandrowska 67/93, 91-205 Łódź, Poland; 3 Department of General Chemistry, Cracow University of Economics, Sienkiewicza 5, 30-033 Kraków, Poland; 4 Department of Food Chemistry and Nutrition, Medical College, Jagiellonian University, Medyczna 9, 30-688 Kraków, Poland

**Keywords:** novel impurities, elemental profile, ICP-MS, health risk assessment, mint tea, tea infusions

## Abstract

The purpose of our studies is the evaluation of the health risks of the novel elemental impurities (Ag, Au, Co, Cs, Li, Mo, Se, Sr, and V) in mint tea infusions (*Mentha piperita* L.) available in Poland. For this purpose, we applied ICP-MS-based methodology for content analysis (elemental profile and µg/L of the infusion) and also the specific health risk assessment for a comprehensive assessment. Our strategy was based on weekly intake (µg/L of infusion/week) considering weekly tea consumption. Then, based on the weekly consumption of tea per adult, individuals were compared to the Temporary Tolerated Weekly Intake (PTWI) designated by the Joint FAO Expert Committee on Food Additives (JECFA), and the weekly consumption per body weight (µg/L of infusion/week/bw) was estimated. Daily exposure to Co in the tested products is in a range of 0.093–0.238 µg/day. In comparison, the established PDE (oral exposure) for Co by the ICH guideline (the ICH guideline Q3D (R1), 2019) is 50 µg/day. For lithium, PDE is approximately 560 µg/day and, in our study, the estimated daily exposure for Li in investigated products is in a range of 0.226–2.41 µg/day. Our studies found also low levels of Cs (in a range of 0.0598–0.195 µg/L), Mo (in a range of 0.663–3.261 µg/L), and Sr (0.223–65.842 µg/L) in infusions. For Molybdenum, the established PDE is approximately 3400 µg/day. There are no documents about Sr and Co in the Joint FAO/WHO Expert Committee on Food Additives (JECFA) database and the ICH guideline Q3D (R1). In the case of V, the established PDE is 120 µg/day, and the level of V in analyzed infusions is in a range of 0.284–0.702 µg/L. Silver and gold were present only in a few samples, and the estimated daily exposure for Ag is approximately 0.04575 µg/day for product A and approximately 0.1885 µg/day for product O, except for Au, which is in a range of 0.000356–0.114 µg/L. The estimated daily exposure for Ag is 167 µg/day and, for Au, it is in a range of 8.9 × 10^5^–0.0285 µg/day. It can be concluded that levels of all investigated elements (novel and also traditional elemental impurities) in the daily dose should not threaten the consumer’s health after consuming mint tea infusions.

## 1. Introduction

For centuries, plant extracts were used by people for different health purposes in different forms, e.g., tinctures, mixtures, and also teas [1]. Teas, in particular, were a convenient and easy form to use in various situations. Among all the known herbs, peppermint is one example of an herb commonly used as a single ingredient in teas or added to multi-ingredient infusions [2]. This kind of tea is often used for health problems with digestive disorders [3]. Although the medicinal properties of herbs are known, the crucial problem from a toxicological point of view is the accumulation of pollutants in the soil where the plant grows. Element ions present in plants are absorbed by the roots, transported to the above-ground parts of plants, and bioaccumulate along with any essential metals present in them, such as Ag, Au, Co, Cs, Li, Mo, Se, Sr, and V [4]. When consumed by plants or their extracts, these pollutants enter the human body, such as tea. Because of the high presence of elemental impurities (EI), it is worth paying attention to them. Some of them are not degradable and may accumulate in the human body and, worse still, may pose a threat to human health [5]. For example, Fe and Cu were found in eighty-seven reported plants, Cl in twenty-three species, and Se and Au in twenty-one and nine plant species [6].

A complex problem for the environment and human health are novel elemental impurities (NEIs). NEIs are not a typical EI (e.g., Co, Mo, Se, Sr, Cs, Li, and V) resulting from current environmental exposure. One of the hottest topics related to NEIs are metallic nanoparticles (especially Ag and Au); however, more is needed to know about their environmental fate, levels, and effects [7]. Excessive consumption of silver can lead to argyria, which is defined as a blue-gray discoloration of the skin due to the deposition of silver [8]. Clinical studies have shown that Au consumed in excess is absorbed into the circulation and metabolized to the liver, kidneys, spleen, lymph nodes, skin and appendages, salivary gland, bones, and bone marrow. Gold consumed in excess does not show toxicity [9]. There are also environmental NEIs that, on the one hand, seem to be of minor importance but generally have never been assessed because of their problematic nature, i.e., Co, Mo, Se, Sr, Cs, Li, and V. Cobalt is essential in the human body because it is an integral component of Vitamin B_12_; however, no particular biological function of cobalt in the human body has been identified [10]. Co compounds (e.g., cobalt octanoate) are being used as catalysts in the selective hydrogenation process which can be a potential source of this element in a habitat [11]. Excessive cobalt intake has been associated with systemic toxicity that manifests as a clinical syndrome with variable neurological, cardiovascular, and endocrine symptoms [12]. The following particular element is Mo because the range between toxicity and deficiency in animals is narrow, and, therefore, careful control of Mo in animal diets is essential [13]. Toxicity associated with excessive molybdenum intake was associated with respiratory symptoms and an increased number of neutrophils and lymphocytes [14]. On the other hand, selenium is an essential trace element for many species, including humans. The most significant toxicity observed with excessive exposure in humans to Se is selenosis, characterized primarily by dermal and neurological effects, including unstable gait and paralysis [15]. In the scientific literature, there is a lack of studies on environmental exposure to this element. A very sophisticated element is also vanadium; despite its iniquitousness in the body, an essential biological role for this element in humans has not been established [16]. High concentrations of vanadium compounds have been shown to be toxic in inhibiting several enzymes, including oxidative phosphorylation and, in humans, acute or chronic vanadium related poisoning affects the respiratory and digestive systems and causes palpitations, exhaustion, depression, and tremors [17]. The extraordinary element is also strontium because it has never been shown to be an essential element, causing death when absent, but radioactive isotopes (especially ^90^Sr)are extremely dangerous for bones [18]. Another intriguing element is lithium applied as a human therapeutic, and extensive human data exists on administering lithium salts in treating mania, bipolar disorder, and recurrent unipolar depression [19]. However, the measurement of lithium concentrations is required during treatment, but the environmental sources of this element are still unknown. There is little evidence of lithium toxicity when taken in excess, but it is associated with an increased risk of decreased urine concentration, hypothyroidism, hyperparathyroidism, and weight gain [20]. Cesium is an interesting but underestimated element because natural Cs exist mainly as ^133^Cs, and is also slightly inclusive of the other eleven main radioactive isotopes that can be noxious to humans [21]. Cesium toxicity is dose-dependent but full knowledge of its acute and chronic toxicity is not available, Cs side effects include hypokalaemia, seizures, cardiac arrhythmias, syncope and cardiac arrest [22]. The justifications for choosing these EI were:

1. Health risk assessment of new and traditional EI (Ag, Au, Co, Cs, Li, Mo, Se, Sr, and V) for the first time in mint tea infusions (*Mentha piperita* L.) available in Polish markets;
2. EI profile in mint tea infusions (µg/L of infusion) as raw results;
3. Estimation of the weekly intake (µg/L of infusion/week) established on weekly tea consumption (based on [23]);
4. Estimation of the weekly intake per body weight (µg/L of infusion/week/bw) based on weekly tea consumption per individual (~70 kg bw) in comparison to the Provisional Tolerable Weekly Intake (PTWI) appointed by the Joint FAO/WHO Expert Committee on Food Additives (JECFA); and
5. Individual health risk assessment for elements without PTWI values.

## 2. Materials and Methods

### 2.1. Samples

Samples of mint tea (*n* = 17) were purchased in stores in Poland in five cities (Rzeszów, Poznań, Warsaw, Gdańsk, and Krakow) from June 2021 to September 2021. The analysed samples were in various forms, such as raw materials (leaf-like/needle-like) and tea bag packages (20–25 pieces of boxes; 1.4 g–2.0 g of raw materials). No additional steps in sample preparation were required. The samples were not subjected to further preparatory steps. They have been applied in our studies based on consumer conditions (tea infusion). The characteristics of the analyzed mint tea samples analyzed are shown in Table 1.

### 2.2. Chemicals

In our studies, we analyzed nine elements: Ag, Au, Co, Cs, Li, Mo, Se, Sr, and V. For this purpose, two multi-element stock solutions (CHECL01.13632.0100 and Merck 1.10580.0100) containing Ag, Au, Co, Cs, Li, Mo, Se, Sr, and V were utilized as an internal standard. Table 2 shows the concentrations of the elements in a multi-element stock solution. Nitric acid (Suprapure grade, 65%) was obtained from Merck (Lowe, NJ, USA).

### 2.3. Apparatus

The determination of investigated elements (Ag, Au, Co, Cs, Li, Mo, Se, Sr, and V) was performed by the methodology based on the ICP-MS technique (Perkin Elmer Elan DRC-e spectrometer (PerkinElmer, Waltham, MA, USA)). We used the simultaneous multielement detection mode. The plasma excitation power was 1150 W; the gas flow rates for plasma gas, carrier gas, and makeup gas were 15.0, 1.1, and 1.0 L min^−1^, respectively. All experimental conditions are summarized in Table 3.

### 2.4. The Procedure of the Study

A tea infusion process was performed based on the information described in Table 1 (the number of raw materials for the infusion process and infusion time). Boiling ultrapure demineralized water (200 mL) was added to a 250 mL Erlenmeyer flask with the appropriate amount of analyzed tea. The tea infusion was then stirred, then covered for 3–8 min (based on the manufacturer’s recommendations) to ensure adequate wetting. After the infusion process, the obtained solutions were decanted and cooled to room temperature until an ICP-MS-based methodology performed the analysis.

**Figure 1:**
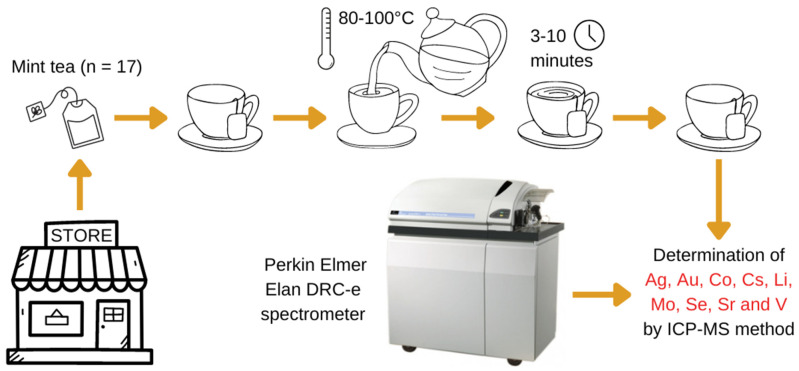
The mint tea infusions process procedure workflow and the determination of investigated elements: Ag, Au, Co, Cs, Li, Mo, Se, Sr, and V.

The determination of the elements was based on contemporary multi-element detection of Ag, Au, Co, Cs, Li, Mo, Se, Sr, and V by an Elan DRC-e Perkin Elmer (US) spectrometer. The determination of elements was based on simultaneous multielement detection of the investigated elements by the Elan DRC-e Perkin Elmer (US) spectrometer. When the infusion process was complete, the obtained solutions were decanted and cooled to room temperature until analyzed by ICP-MS. The workflow of our study is shown in Figure 1.

### 2.5. The Calibration and Quality Control

The determination of the investigated elements in the analyzed samples was performed using the calibration curves (by diluting a stock standard of the tested elements in a range of 1:100–1:10,000). The working solutions (*n* = 5) with concentrations of Ag, Au, Cs, and Sr were 0.0, 1.0, 2.0, 5.0, and 10.0 µg/L, and for Co, Li, Mo, and V, they were 0.0, 2.0, 4.0, 10.0, and 20.0 µg/L. Additionally, for Se, the concentrations were 0.0, 10.0, 20.0, 50.0, and 100.0 µg/L. Then, solutions of the predetermined concentrations were applied for the calibration procedure. The analysis was precise and accurate, as indicated by the values of the correlation coefficient (0.995 < R < 0.999). Table 4 summarizes the analytical calibration strategy and the quality control results.

### 2.6. Health Risk Assessment

An estimation of the health hazard of investigated EI in mint teas from Polish markets involves three steps:

1. The tea infusion (µg/L of infusion) using elemental impurity profile, the half violin plots, and the descriptive statistics (minimum, maximum, and mean);
2. The assessment of weekly intake (µg/L of infusion/week) established on weekly tea consumption (about 6 L of tea per week, based on [23]), as seen in Equation (1):

EWI = RR × 6 (L/week)(1)

where EWI is the estimated weekly intake [µg/L of infusion/week] and R is the raw results [µg/L]; and

3. Based on the weekly tea consumption per individual (at about 70 kg/bw) in comparison to the PTWI. The weekly intake was evaluated depending on body weight (µg/L of infusion/week/bw), as seen in Equation (2):

EWIBW = EWI/BW(2)

where EWI is the estimated weekly intake [µg/L of infusion/week] and BW is the average body weight (approximately 70 kg bw) [kg].

### 2.7. Statistical Analysis

Excel macros (Excel 2010; Microsoft Office, Rzeszow, Poland) were applied for initial data collection. All data were expressed as means of five independent replicates with the standard error (the mean ± standard error). Descriptive statistics were made using the Origin 2021 Pro program (licensed by Jagiellonian University): minimum, maximum, and mean. Moreover, utilizing the same program, EI profiles were created as half-violin charts for each element. Each stage of the health risk assessment approach was calculated for self-programmed macros in Excel (Excel 2010; Microsoft Office) and the Origin 2021 Pro program (licensed by Jagiellonian University).

## 3. Results and Discussion

### 3.1. NEIs and Traditional Impurities Profile in Analysed Mint Tea Samples

**Figure 2:**
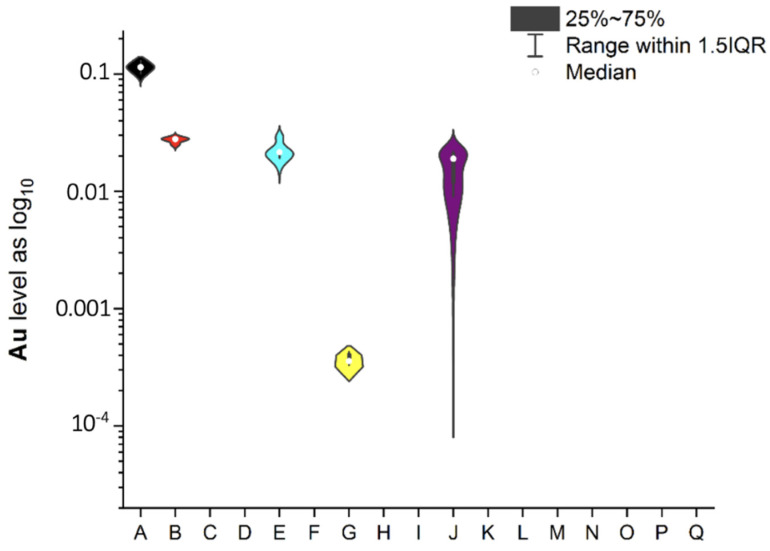
The plot as a violin with the box for the Au level in a log_10_ scale in examined products. (i.e., A, B, E, G, and J).

**Figure 3:**
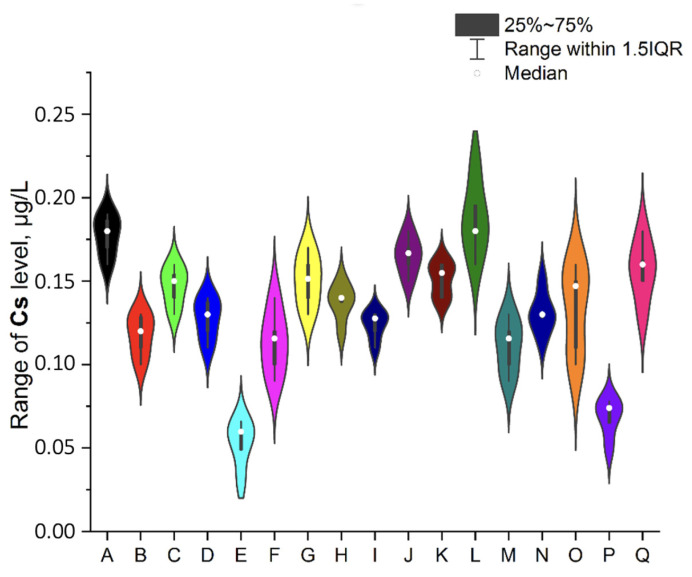
The plot as a violin with the box for the Cs level (µg/L) in the examined products (A–Q).

**Figure 4:**
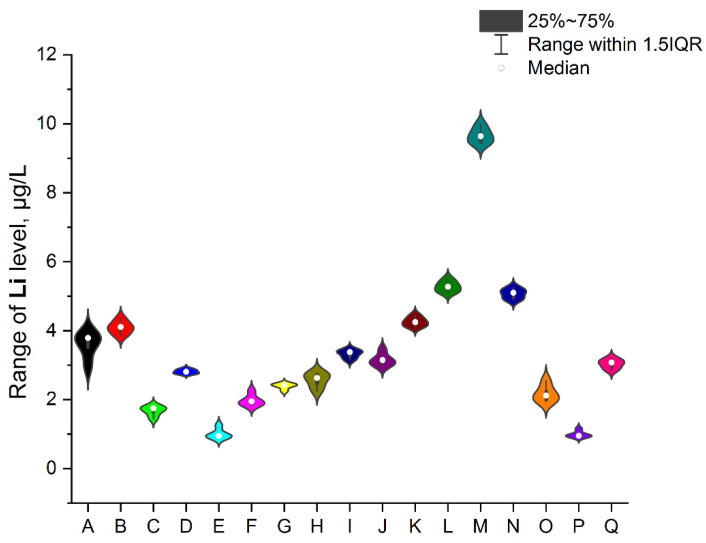
The plot as a violin with the box for the Li level (µg/L) in the examined products (A–Q).

**Figure 5:**
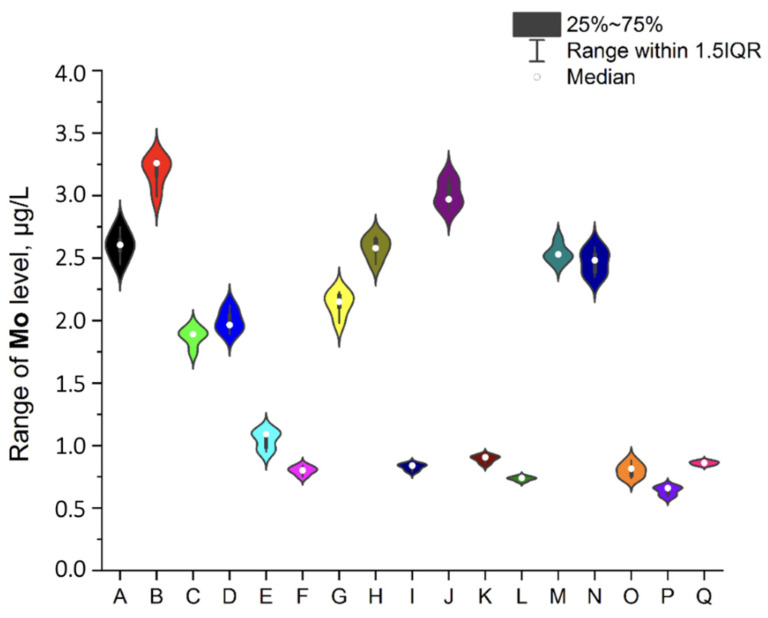
The plot as a violin with the box for the Mo level (µg/L) in the examined products (A–Q).

**Figure 6:**
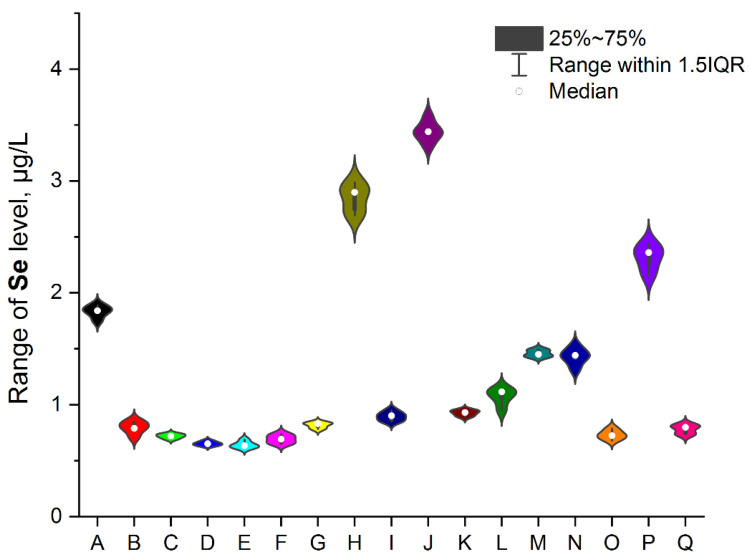
The plot as a violin with the box for the Se level (µg/L) in the examined products (A–Q).

**Figure 7:**
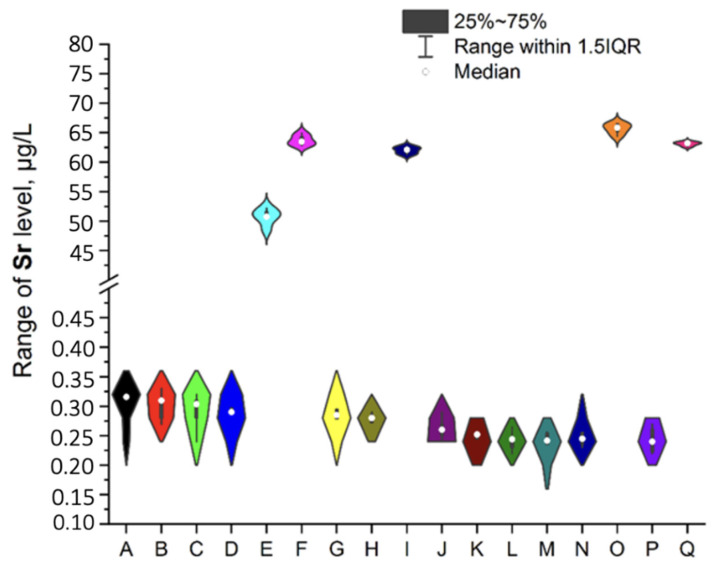
The plot as a violin with the box for the Sr level (µg/L) in the examined products (A–Q).

**Figure 8:**
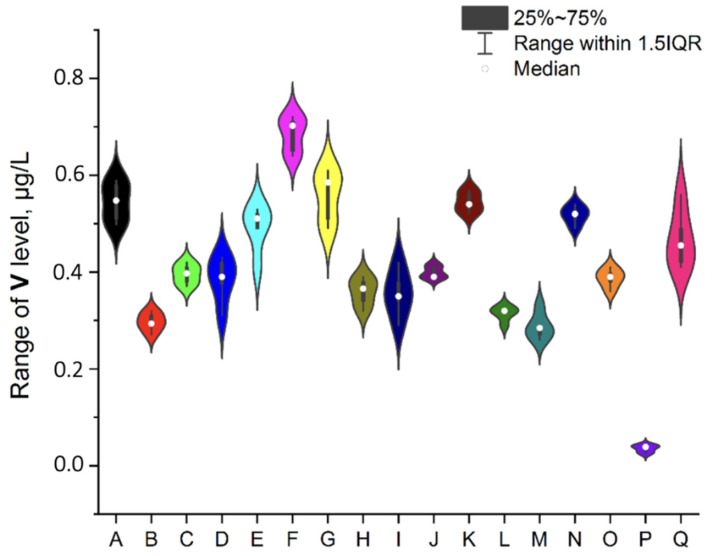
The plot as a violin with the box for the V level (µg/L) in the examined products (A–Q).

To ensure better transparency and more precise analysis of each EI separately (without Ag due to the deficient levels in two samples, i.e., A and O), the plots as a violin with the box were prepared (Figure 2, Figure 3, Figure 4, Figure 5, Figure 6, Figure 7 and Figure 8). Additionally, the descriptive statistics (minimum, maximum, mean, and RSD) are shown in Table 5.

All investigated elemental impurities were present in all analyzed mint tea infusions, except silver and gold. Ag was present only in two samples (A, mean: 0.754 ± 0.08 µg/L and O, mean: 0.183 ± 0.06 µg/L). Au was present in five samples (A, B, E, G, and J) at shallow levels (in a range of 0.000356–0.114 µg/L). Four EI were characterized by levels below 1 µg/L (i.e., Cs: 0.078–0.186 µg/L; V: 0.039–0.702 µg/L and Co: 0.372–0.952 µg/L). Additionally, four EI was characterized by levels below 10 µg/L (i.e., Mo: 0.663–2.970 µg/L, Se: 0.622–3.417 µg/L, and Li: 0.904–9.641 µg/L). The relatively high and variable levels of EI were observed for Cr and Sr (Cr in a range of 7.317–23.451 µg/L and Sr in a range of 0.223–65.842 µg/L). The lower level was noted for Au (3.6 × 10^−4^ µg/L in sample G). It should be underlined that the highest levels of EI were observed for Sr in five samples: E (50.711 ± 0.87 µg/L), F (64.270 ± 0.98 µg/L), I (62.103 ± 1.12 µg/L), O (65.841 ± 0.94 µg/L), and Q (63.263 ± 1.11 µg/L). These observations are not correlated with tea-related factors (form, amount of raw material for infusion, brew time, or country of origin). On the other hand, Sr levels were relatively low in other samples (in a range of 0.223 µg/L–0.309 µg/L).

As was mentioned in the introduction, only one article described by [24] is related to the determination of selected elements (Ba, Ca, Co, Cu, Fe, I, Li, Mg, Mn, Ni, Se, Sn, Sr, Ti, V, and Zn) in peppermint infusions. The comparison of the obtained values (means) with the literature data [24] is presented for selected elements in Table 6.

The analysis and comparison of our data (means, (µg/L) with the literature values (from [24], mg/kg) indicated that the obtained values are significantly lower (approximately 30–125 times) than the literature data. The most considerable discrepancies were observed for V (our results/literate results: 1/270). Such differences probably result from the comparison of average values for our results characterized by variable ranges and the number of investigated products (*n* = 17) compared with only a few samples (*n* = 6), based on the literature [24].

### 3.2. The Assessment of the Weekly Exposure in Comparison with the Weekly Intake of Tea

For the health risk assessment of investigated EI, the second step is the assessment of EI weekly exposure in comparison with the weekly intake of mint tea (based on consumption scenarios). It is difficult because there are a lot of consumption scenarios. The worst-case (WC) scenario is usually applied in toxicological risk assessment, i.e., in this case, the highest possible frequency of tea consumption per week. Based on the assumptions of [23], that the average consumer drinks six teas per week, the estimation of the weekly intake of EI in mint tea infusions are presented in Table 7. Due to the lack of any toxicological reference values for this analysis, the debate for this part is impossible; however, this step (i.e., multiplying obtained values six times) is essential for the final stage of the health risk assessment and the estimation of weekly intake relying on body weight established on weekly mint tea consumption.

### 3.3. The Assessment of the Weekly Intake of Each Element, including Body Weight Based on Weekly Consumption

The last step in the appropriate health risk assessment of investigated elements in mint tea infusions is the assessment of weekly intake based on weight and weekly tea consumption. For this purpose, the values of the weekly intake for each EI in investigated samples (Table 6) were calculated by dividing by 70 kg (average adult human weight, recommended by EFSA [25]). The obtained results are presented in Table 8.

An appropriate baseline (reference values) for the final step in the applied health risk assessment is the comparison of the obtained estimation of weekly intake depending on body weight with the values of the provisional tolerable weekly intake. This widely applied parameter in assessing limitations for redundant elements in the diet is issued by JECFA [26,27]. The idea of PTWI was established in 1972 by JECFA as an intake value expressed weekly, usually presented as µg of pollutant per week per 1 kg of body weight. It should be underlined that this idea emphasizes the significance of long-dated exposure that is not expressed by another point of reference, i.e., provisional maximum tolerable daily intake (PMTDI); it is analog but on a daily basis. Nevertheless, evaluations of the JECFA include only a few investigated EI, i.e., only heavy metals (PTWI_As_ = 0.015 mg/kg bw/week [28], PTWI_Cd_ = 7.0 µg/kg bw/week [29], PTWI_Pb_ = 0.025 mg/kg bw/week [30], and selenium (PTWI_Se_ = 66.0 µg/kg bw/week [31]). For this goal, the proportions of the obtained values of weekly intake (µg/kg bw/week) for the selected EI to the established PTWI were calculated and presented in Table 9.

The results in Table 9 demonstrate that the weekly exposure for Se compared to PTWI is generally low (it does not exceed 1% in any case) for Se, in a range of 0.08–0.49%.

### 3.4. Health Risk Assessment of NEIs in Analysed Teas

Due to the lack of established PTWI values for NEIs (Ag, Au, Co, Cs, Li, Mo, Sr, and V), the individual health risk assessment, including all available information, has been described below.

#### 3.4.1. Silver

Given the rare application of Ag and in the absence of knowledge of the exact nature of this element in food, specification and any toxicological reference values were not prepared by JECFA since 1978 [32] until now (not re-evaluated by JECFA, but the provisions for Ag was withdrawn at 50th meeting of the Codex Alimentarius Committee on Food Additives, CCFA50). Hence, evaluating weekly exposure to Ag in mint tea infusion is impossible. Additionally, in the scientific literature, there is a lack of articles about this element’s content in mint tea infusion. The obtained results show that silver was present only in two samples (A and O) at relatively low levels (0.183 µg/L and 0.754 µg/L for samples A and O, respectively). Including the daily consumption (about 250 mL of mint tea infusion per day), the estimated daily exposure for Ag is approximately 0.04575 µg/day for product A and approximately 0.1885 µg/day for product O.

The established permitted daily exposure (PDE and oral exposure) for Ag by the ICH guideline Q3D (R1) on elemental impurities [33] is 167 µg/day. Hence, the obtained results indicated 0.028% and 0.113% of the established oral PDE values for samples A and O, respectively. It can be concluded that Ag impurities are not a severe problem for human health and environmental points of view. The obtained results may be valuable for other investigators due to the lack of similar studies.

#### 3.4.2. Gold

As was the case with Ag, any toxicological reference values were not prepared for Au by the Joint FAO/WHO Expert Committee on Food Additives (JECFA) since 1978 [34] until now (not re-evaluated by JECFA, but the provisions for Au were withdrawn at 50th meeting of the Codex Alimentarius Committee on Food Additives, CCFA50). Additionally, a literature review indicated a lack of studies related to this topic. In our studies, we found that Au was present only in five samples (A, B, E, G, and J) at deficient levels, i.e., <0.2 µg/L (in a range of 0.000356–0.114 µg/L). The estimated daily exposure for Au (about 250 mL of mint tea infusion per day) is in a range of 8.9 × 10^−5^–0.0285 µg/day. In comparison to the established PDE (oral exposure) for Au by the ICH guideline Q3D (R1) [33] of 134 µg/day, Au impurities are not hazardous for human health, and it is not a pollutant with a crucial concentration in the environment.

#### 3.4.3. Cobalt

As was mentioned in the introduction, Co is an essential element for humans because it is an integral component of Vitamin B_12_; however, no particular biological function of this element in the human body has been identified [11]. There is no evaluation of Co in the Joint FAO/WHO Expert Committee on Food Additives (JECFA) database. The estimated daily exposure for this element in investigated products (about 250 mL of mint tea infusion per day) is in a range of 0.093–0.238 µg/day. In comparison to the established PDE (oral exposure) for Co by the ICH guideline Q3D (R1) [33] of 50 µg/day, Co impurities are shallow (0.186% and 0.476% of established oral PDE). Hence, Co impurities are not a potential problem for humans, including drinking mint tea infusions available in Poland.

#### 3.4.4. Cesium

Cesium is an interesting but underestimated element because natural Cs mainly exist as ^133^Cs. It is also slightly inclusive of the other eleven major radioactive isotopes, which can be noxious for humans [21]. Our studies found low levels of this element in infusions (in a range of 0.0598–0.195 µg/L). There are no documents about Cr in the Joint FAO/WHO Expert Committee on Food Additives (JECFA) database and the ICH guideline Q3D (R1). Additionally, there are no available articles related to this topic in the scientific literature (except studies about the association of ^137^Cs with various components of tea leaves fermented from Chernobyl-contaminated green tea; however, there are no values for Cs level in infusion). Hence, it is impossible to compare the obtained results, but this element’s low levels indicate no severe problems for human health.

#### 3.4.5. Lithium

Lithium is a common element in the environment but is more often applied (Li salts) as a pharmaceutical in treating mania, bipolar disorder, and recurrent unipolar depression rather than in industry. In general, there is a deficiency of studies related to this element about existence in herbs or herbs infusions. The established PDE (oral exposure) for Li by the ICH guideline Q3D (R1) [33] is based on human experience with this element and is approximately 560 µg/day. The estimated daily exposure for Li in investigated products (about 250 mL of mint tea infusion per day) is in a range of 0.226–2.41 µg/day; hence the potential problem with Li exposure after min tea drinking does not exist.

#### 3.4.6. Molybdenum

Mo is an interesting element because the range between toxicity and deficiency in animals is narrow; therefore, careful control of Mo in animal diets is essential [13,34]. From a regulatory point of view, there is only a PDE (oral) value available for this element, i.e., 3400 µg/day [33]. Because the level of Mo in analyzed infusions is in a range of 0.663–3.261 µg/L, there is no hazard for mint tea infusion drinking.

#### 3.4.7. Strontium

Sr is an extraordinary element because it has never been shown to be essential, but radioactive isotopes (especially ^90^Sr) are highly hazardous for bones [18]. However, there are no documents about Sr in the Joint FAO/WHO Expert Committee on Food Additives (JECFA) database and the ICH guideline Q3D (R1). A comparison of obtained results with data described by [24] is discussed earlier (see Section 3.1, Table 5).

#### 3.4.8. Vanadium

Vanadium is a very sophisticated element because it is ubiquitous in the body, but an essential biological role for this element in humans has not been established [16]. There are no opinions about this element in the JECFA database. The established PDE (oral) value described in the ICH guideline Q3D (R1) [33] is 120 µg/day. Since the level of V in analyzed infusions is relatively low (in a range of 0.284–0.702 µg/L), these impurities are not a potential problem for humans, including drinking mint tea infusions available in Poland.

## 4. Conclusions

The level of investigated EI in all of the investigated mint tea infusions occurs but at a relatively low level. The obtained elemental profiles in mint tea infusions indicated the presence of Co (0.372–0.952 µg/L), Cs (0.078–0.186 µg/L), Li (0.904–9.641 µg/L), Mo (0.663–2.970 µg/L), Se (0.622–3.417 µg/L), Sr (0.223–65.842 µg/L), and V (0.039–0.702 µg/L) in all investigated samples. There was a lack of Ag impurities (15/17) and Au impurities (12/17) in most investigated samples. Comparing the obtained results with the literature values [24] indicated that our results are lower (approximately 30–125 times) than the existing literature values. The observed differences probably result from the comparison of average (mean) values for our results characterized by variable ranges and the number of investigated products (*n* = 17) compared to only a few samples (*n* = 6) from the literature [24].

The health risk assessment for Se in the analyzed products indicated no health hazard to consumers for weekly exposure. Due to the lack of established PTWI values for other investigated EI (Ag, Au, Co, Cs, Li, Mo, Sr, and V), the individual health risk assessment, including all available information, has been made. The obtained results indicated that EI levels in daily doses should not represent any health risk to the recipient after consuming mint tea infusions from products accessible in Polish markets.

Our designed health risk assessment strategy for investigated EI provides pioneer data (especially for Ag, Au, Co, Cs, Li, Mo, and V) may be worthwhile for additional investigators and manufacturers. Additionally, a well-design health risk assessment methodology will be valuable and essential for public health and environmental studies. Because these environmental investigations are scarce, it would be valuable to carry out a broader study considering other mint tea infusions in different countries.

## Figures and Tables

**Table 1:** The characteristics of investigated mint tea samples.

Code of Sample	Form	Number of Raw Materials for the Infusion Process, g	Time of the Infusion Process (Brew Time), Minutes	Country of Origin	EAN
A	Tea bag	1.5	8–10	Poland	5906881826065
B	Leaf-like	2.0	7–9	Poland	nd *
C	Tea bag	1.0	5–10	nd *	5900738000103
D	Tea bag	2.0	5–10	Malaysia	5051898696337
E	Tea bag	2.0	10	Poland	5900956002309
F	Tea bag	1.5	5–8	Poland	5900675006275
G	Tea bag	2.0	5	Poland	5901483060206
H	Nylon tea bag	2.0	8–10	Poland	5900396019783
I	Needle-like	1.0–1.5 (teaspoon)	10	Egypt	5909000519206
J	Tea bag	2.0	8	Poland	4056489007159
K	Tea bag	2.0	5-8	Poland	5903886172371
L	Tea bag	1.5	5–8	Poland	nd *
M	Silk tea bag	2.0	10	Poland	5909990029303
N	Needle-like	1.0–1.5 (teaspoon)	5–8	Poland	5901483130022
O	Tea bag	1.5	3–5	Poland	5900175431737
P	Needle-like	1.0–1.5 (teaspoon)	10	Poland	5900956006628
Q	Tea bag	1.4	5–7	Poland	5900888010915

* nd—no data.

**Table 2:** The concentration of each analyzed element in an applied multi-element stock solution.

Element	Concentration in an Applied Multi-Element Stock Solution, mg·L^−1^
Ag	10.0
Au	10.1
Co	20.0
Cs	10.0
Li	19.8
Mo	19.9
Se	101.0
Sr	9.5
V	20.0

**Table 3:** The adjusted operational requirements of an applied ICP-MS apparatus.

ICP-MS	Elan DRC-e Perkin Elmer (US)
Sample introduction	Scott spray chamber
Skimmer cone	Ni
Sampler cone	Ni
RF power, W	1150
Number of sweeps/readings	4
Number of readings/replicates	2
Number of replicates	3
Cooling gas flow rate(L·min^−1^)	17
Scanning mode	Peak hopping

**Table 4:** The summary of the analytical calibration strategy and quality control results.

Analyte	Calibration Function		R	Recovery, %
	Sigma A	Slope		
Ag	49.3241	3141.38	0.99986	98 ± 1.0
Au	4.45668	69.3612	0.99586	97 ± 1.3
Co	10.066	8651.94	0.99999	99 ± 0.7
Cs	46.851	14,413.5	0.99988	97 ± 1.5
Li	3.80757	1006.25	0.99998	98 ± 1.1
Mo	23.9563	3380.03	0.99992	97 ± 1.5
Se	0.79798	153.457	0.99996	97 ± 1.3
Sr	115.175	16,393.8	0.99992	96 ± 1.6
V	11.3286	7996.7	0.99999	98 ± 1.2

**Table 5:** The descriptive statistics of examined EI in each analyzed sample (A–Q).

Element	Minimum, μg/L	Maximum, μg/L	Mean, μg/L	RSD, %
Ag	0.183	0.754	0.469	1.8–3.94
Au	0.000356	0.114	0.0350	2.37–4.17
Co	0.372	0.952	0.579	0.8–6.8
Cs	0.0598	0.195	0.138	1.1–4.3
Li	0.904	9.641	3.353	0.1–9.64
Mo	0.663	3.261	1.720	0.4–6.4
Se	0.693	3.417	1.307	1.9–2.84
Sr	0.223	64.270	18.202	0.7–5.7
V	0.284	0.702	0.420	1.5–10.34

**Table 6:** The comparison of the obtained results (means, (µg/L) with the literature data [24] for Co, Li, Se, Sr, and V in the analyzed samples.

Element	Obtained Mean Value	
	Obtained Mean Value, µg/L	Literature Values (Łozak et al., 2002), mg/kg
Co	0.579 ± 0.062	0.056 ± 0.001
Cr	13.779 ± 0.95	0.390 ± 0.019
Li	3.353 ± 0.36	0.339 ± 0.008
Se	1.307 ± 0.12	0.087 ± 0.033
Sr	18.202 ± 0.85	2.26 ± 0.06
V	0.420 ± 0.032	0.113 ± 0.006

**Table 7:** The assessment of weekly intake of the investigated elements based on mint tea consumption.

Product	Assessment of Weekly Intake, µg/Week								
	Ag	Au	Co	Cs	Li	Mo	Se	Sr	V
A	236.401	0.686	5.712	1.119	22.738	15.637	11.174	1.895	3.286
B	-	0.163	3.565	0.772	24.839	19.569	4.728	1.858	1.761
C	-	-	4.152	0.924	9.732	11.298	4.419	1.820	2.383
D	-	-	2.913	0.824	16.860	11.788	3.914	1.783	2.256
E	-	0.129	2.351	0.359	5.426	6.547	3.735	304.267	3.064
F	-	-	2.657	0.694	11.765	4.809	4.160	385.622	4.214
G	-	0.002	3.663	0.909	14.314	13.255	5.011	1.672	3.510
H	-	-	2.756	0.825	15.792	15.943	17.387	1.635	2.195
I	-	-	2.550	0.766	20.328	4.882	5.408	372.623	2.271
J	-	0.071	3.206	1.001	18.157	17.822	20.503	1.561	2.336
K	-	-	3.969	0.929	25.904	5.443	5.586	1.524	3.372
L	-	-	4.372	1.171	31.651	4.381	6.692	1.487	1.925
M	-	-	2.915	0.694	57.845	15.177	8.569	1.449	1.707
N	-	-	4.743	0.769	30.619	14.895	8.650	1.412	3.169
O	10.801	-	2.233	0.883	11.855	4.890	4.345	395.050	2.388
P	-	-	4.588	0.468	5.724	3.975	14.190	1.338	0.234
Q	-	-	2.737	0.975	18.477	5.171	4.792	379.579	2.730

**Table 8:** The weekly intake per body weight estimation is based on weekly mint tea consumption (six infusions per week).

Product	Estimation of Weekly Intake, µg/Week/bw								
	Ag	Au	Co	Cs	Li	Mo	Se	Sr	V
A	3.377	0.010	0.082	0.016	0.325	0.223	0.160	0.027	0.047
B	-	0.002	0.051	0.011	0.355	0.280	0.068	0.027	0.025
C	-	-	0.059	0.013	0.139	0.161	0.063	0.026	0.034
D	-	-	0.042	0.012	0.241	0.168	0.056	0.025	0.032
E	-	0.002	0.034	0.005	0.078	0.094	0.053	4.347	0.044
F	-	-	0.038	0.010	0.168	0.069	0.059	5.509	0.060
G	-	0.000	0.052	0.013	0.204	0.189	0.072	0.024	0.050
H	-	-	0.039	0.012	0.226	0.228	0.248	0.023	0.031
I	-	-	0.036	0.011	0.290	0.070	0.077	5.323	0.032
J	-	0.001	0.046	0.014	0.259	0.255	0.293	0.022	0.033
K	-	-	0.057	0.013	0.370	0.078	0.080	0.022	0.048
L	-	-	0.062	0.017	0.452	0.063	0.096	0.021	0.027
M	-	-	0.042	0.010	0.826	0.217	0.122	0.021	0.024
N	-	-	0.068	0.011	0.437	0.213	0.124	0.020	0.045
O	0.154	-	0.032	0.013	0.169	0.070	0.062	5.644	0.034
P	-	-	0.066	0.007	0.082	0.057	0.203	0.019	0.003
Q	-	-	0.039	0.014	0.264	0.074	0.068	5.423	0.039

**Table 9:** The percentages (%) of the acquired values for Se of weekly consumption (µg/kg bw/week) to the established PTWI.

Product	The Ratios (%) of the Obtained Values of Weekly Intake (µg/kg bw/week) to the PTWI for Each Element
A	0.24
B	0.10
C	0.10
D	0.08
E	0.08
F	0.09
G	0.11
H	0.38
I	0.12
J	0.44
K	0.12
L	0.14
M	0.19
N	0.19
O	0.09
P	0.31
Q	0.10

## Data Availability

The datasets generated during and/or analyzed during the current study are available from Kamil Jurowski (kjurowski@ur.edu.pl) upon reasonable request.
